# MEMS ultrasonic transducers for safe, low-power and portable eye-blinking monitoring

**DOI:** 10.1038/s41378-022-00396-w

**Published:** 2022-06-13

**Authors:** Sheng Sun, Jianyuan Wang, Menglun Zhang, Yuan Ning, Dong Ma, Yi Yuan, Pengfei Niu, Zhicong Rong, Zhuochen Wang, Wei Pang

**Affiliations:** 1 State Key Laboratory of Precision Measuring Technology and Instruments, Tianjin University, Tianjin, China; 2 Department of Computer Science and Technology, University of Cambridge, Cambridge, UK

**Keywords:** Electrical and electronic engineering, Environmental, health and safety issues

## Abstract

Eye blinking is closely related to human physiology and psychology. It is an effective method of communication among people and can be used in human–machine interactions. Existing blink monitoring methods include video-oculography, electro-oculograms and infrared oculography. However, these methods suffer from uncomfortable use, safety risks, limited reliability in strong light or dark environments, and infringed informational security. In this paper, we propose an ultrasound-based portable approach for eye-blinking activity monitoring. Low-power pulse-echo ultrasound featuring biosafety is transmitted and received by microelectromechanical system (MEMS) ultrasonic transducers seamlessly integrated on glasses. The size, weight and power consumption of the transducers are 2.5 mm by 2.5 mm, 23.3 mg and 71 μW, respectively, which provides better portability than conventional methods using wearable devices. Eye-blinking activities were characterized by open and closed eye states and validated by experiments on different volunteers. Finally, real-time eye-blinking monitoring was successfully demonstrated with a response time less than 1 ms. The proposed solution paves the way for ultrasound-based wearable eye-blinking monitoring and offers miniaturization, light weight, low power consumption, high informational security and biosafety.

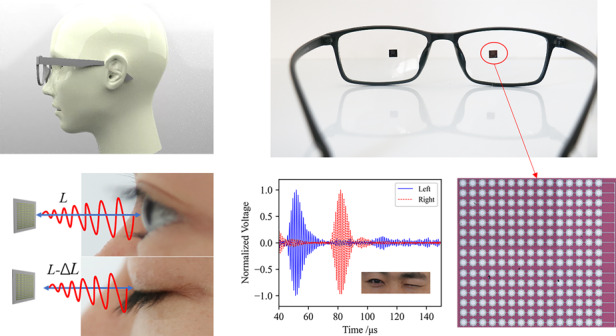

## Introduction

Eye-blinking is one of the most natural and frequent human activities. A person usually blinks 15–20 times per minute on average, approximately 10,000 times per day^1^. Eye-blinking behavior can be classified into unconscious blinking and conscious blinking. The frequency and duration of unconscious blinks are closely related to our psychological and physiological activities^2^. For example, when a person is tired, the rate of blinking increases from an average of one blink every 2–6 s to an average of 1–2 blinks per second, and the time of closing the eyes changes from 0.2 s under normal conditions to more than 0.8 s. For example, tired driving is one of the main factors of traffic accidents. Monitoring the eye-blinking activity of drivers in real time can detect whether a driver is in a fatigued driving state, thereby avoiding the occurrence of traffic accidents^3^. During focused reading, blinking usually occurs at the end of each sentence. Therefore, monitoring a reader’s eye-blinking activity can determine whether the reader is in a state of focused reading. Blinking is also related to the rehabilitation of Bell’s palsy patients. Based on the frequency and time of blinking, the recovery status of the patients can be evaluated^4^.

Conscious blinking is also a promising research field of interest. Blinking activity can be used for simple personal communication. For example, when soldiers are on the battlefield or hidden quietly, informative blinking can help them interact with others without speaking or hand gesturing. Blinking can also provide new communication methods for patients who are unable to speak due to physical damage or when wearing a ventilator. Eye blinking can provide a simple method of interaction between patients and doctors. In addition, blinking monitoring can be adopted in human–machine interactions. For example, by regarding an eye-blinking event as a mouse click, computers can take input from disabled people or patients with disabilities^5^. Furthermore, people are able to experience immersive interaction with AR/VR headsets and smart glasses by seamlessly integrating eye-blinking monitoring and eye-tracking functions into smart devices.

Current available blink monitoring methods include video-oculography (VOG)^6^, electro-oculograms (EOGs)^7^ and infrared oculography (IROG)^8,9^. VOG requires integrating a camera to capture eye images, thereby incurring high computational power and suffering from low image quality under extremely strong light/dark environments. Furthermore, the long-term use of cameras to record human head information may infringe on privacy. An EOG is robust to environmental variations, but the sensor must be attached to the skin near the eye during the test. As a result, wearing the EOG sensor for a long time may cause discomfort, which requires more comfortable and portable solutions. In contrast, IROG does not have the above issues. However, after long exposure to infrared rays, delicate eyes are prone to dryness and fatigue, with safety risks and potential permanent damage when high-intensity infrared is used^10^.

Ultrasound is commonly used in medical imaging, industrial testing and ranging. Sonar is now the most common method of underwater detection and underwater communication. Ultrasound can also work in extreme environments and is not affected by light conditions. It does not extract human facial information if only ranging data are gathered by ultrasound, so privacy is guaranteed. Most importantly, ultrasound has been widely applied in eyeball imaging and does not cause eye injury, thus offering excellent biological safety.

To the authors’ knowledge, very limited eye-blinking monitoring research work has been done using ultrasound^11^. Therefore, we explored the potential of using ultrasound to monitor eye-blinking activity. However, even though conventional ultrasonic probes are widely used, they are usually heavy and large in size and therefore not suitable for portable applications. For long-term monitoring, new ultrasonic probes and systems should be developed as a more comfortable and portable solution. With the development of microelectromechanical system (MEMS) technology, MEMS ultrasonic transducers with small footprints and light weight have been investigated^12–16^.

In this paper, we developed a portable system to monitor eye-blinking activity using low-intensity ultrasound. To achieve a small size and light weight, miniaturized and low-power MEMS ultrasonic transducers were designed, fabricated and seamlessly integrated into glasses. Since the MEMS ultrasonic transducer was only millimeters in size, user-friendly glasses were implemented for real-time monitoring experiments of eye-blinking activity. Based on time-of-flight (TOF) pulse echoes and a dynamic unsupervised learning method, we achieved eye state recognition as a demonstration of portable human blink monitoring.

## Design and fabrication

### Transducer element design

The MEMS ultrasonic transducer developed in this study uses aluminum nitride (AlN) as the piezoelectric thin film for ultrasound generation and detection. In comparison with the commonly used PZT, AlN has the advantages of higher receiver sensitivity, easier integration with a complementary metal oxide semiconductor (CMOS) circuit, and nontoxicity compared to other materials, such as PZT, which contains poisonous Pb, making AIN especially suitable for portable applications.

**Fig. 1:**
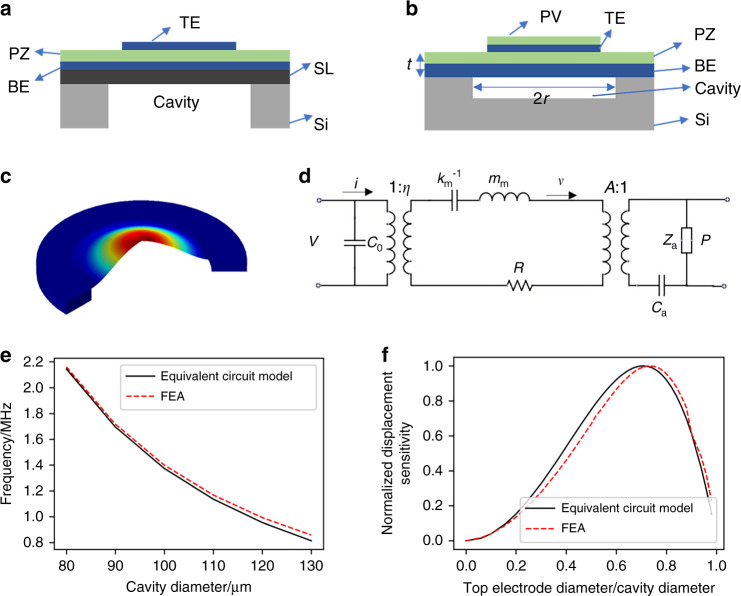
Design of a single MEMS ultrasonic transducer. **a** The common MEMS ultrasonic transducer structure. **b** Structure schematic of the MEMS ultrasonic transducer in this work. **c** A0 bending vibration mode of the MEMS ultrasonic transducer. **d** Equivalent circuit model of a single MEMS ultrasonic transducer. **e** Relationship between the resonant frequency and the cavity diameter in air from the results of the equivalent circuit model and finite element analysis (FEA). **f** Relationship between the ratio of the electrode diameter to the cavity diameter and the normalized displacement sensitivity from the results of the equivalent circuit model and FEA

The commonly used piezoelectric MEMS ultrasonic transducer is generally composed of a support layer (SL), bottom electrode (BE), piezoelectric layer (PZ) and top electrode (TE), and it has a back-etching cavity^17^, as shown in Fig. 1a. The MEMS ultrasonic transducer in this work comprises four thin-film layers: molybdenum (Mo) as the bottom electrode, AlN as the piezoelectric layer, Mo as the top electrode and AlN as the passivation layer (PV), as shown in Fig. 1b. The transducer structure does not include the support layer but instead uses the thickened Mo electrode to adjust the neutral axis of the entire vibrating structure. Since the transducer structure does not require the support layer, expensive SOI silicon wafers are also not needed in fabrication. In addition, no additional SiO_2_^18^ or AlN^19^ film is needed as the support layer, simplifying the laminated structure and reducing the fabrication complexity.

Meanwhile, the transducer in this work adopts a front-etching cavity structure rather than a back-etching cavity. The front-etching cavity leads to a sealed and well-controlled cavity compared to the back-etching cavity. As back etching usually leads to undercuts by a deep reactive ion etching (DIRE) process, the fabricated cavity diameter is hard to control and usually deviates from the design, especially when the silicon substrate is thick, e.g., 400 μm. Thus, the undercut leads to inconsistency in the resonant frequencies from transducer element to element and transducer array to array. Because the front etching cavity is shallow and etched by a reactive-ion etching (RIE) process instead of DIRE, the process is simpler, and the cavity dimension control is more accurate. Based on our design, the shallow cavities have an average diameter of 120 μm with an error of only 0.3 μm, leading to good control of the transducer element and array.

When alternating voltage is applied to the top and bottom electrodes, the piezoelectric layer generates transverse stress due to the inverse piezoelectric effect, thereby exciting bending vibration of the structured films (Fig. 1c). The resonant frequency of the MEMS ultrasonic transducer is determined by the cavity radius *r* and the thickness of the structure *t*^20^.

1 $$F \propto \sqrt {\frac{{k_{{{\mathrm{m}}}}}}{{m_{{{\mathrm{m}}}}}}} \propto \frac{t}{{r^2}}$$

where *k*_m_ is the equivalent stiffness and *m*_m_ is the equivalent mass of the first-order mode in the field of mechanics.

We established an equivalent circuit model for analytical analysis, as shown in Fig. 1d. In this model, *C*_0_ represents the static capacitance of the MEMS ultrasonic transducer, *R* is the mechanical loss in the device, *A* is the effective surface area of the structure, *C*_a_ is the acoustic compliance of the cavity, *η* is the electromechanical coupling coefficient, and *Z*_a_ is the acoustic impedance.

When the circular plate is in the first-order A0 vibration mode, the equivalent stiffness, equivalent mass and electromechanical coupling coefficient of the mechanical module are given by^21^

2 $$k_m = \frac{{64\pi D}}{{3r^2}}$$

3 $$m_m = \frac{{\pi r^2\mu }}{5}$$

4 $$\eta = 4\pi \gamma ^2(\gamma ^2 - 1)e_{31,f}\bar Z_p$$

where *D* is the flexural stiffness of the structure, *μ* is the mass per unit area, *r* is the radius of the cavity, *γ* is the ratio of the top electrode radius to the radius of the cavity, and $\bar Z_p$ is the distance from the center of the piezoelectric layer to the neutral axis.

In the acoustic field, the load acoustic impedance^22^ and the cavity acoustic compliance^23^ are

5 $$Z_a = \frac{{\rho c}}{A}\left( {R_r + jX_r} \right)$$

6 $$C_a = \frac{{\pi r^2h}}{{\rho c^2}}$$

where *ρ* is the air density, *c* is the propagation speed of sound waves in the air, and *h* is the depth of the cavity.

The current in the electrical domain refers to the velocity *v* at the center of the transducer, and the current is measured by a current probe. Then, we can calculate the displacement *d* by

7 $$d = \frac{v}{{2\pi f}}$$

In most cases, the distance between the glasses and the eyeball is approximately 9 mm to 16 mm. The working frequency of the MEMS ultrasonic transducer is designed as 1 MHz. If the frequency is much lower, the axial detection resolution will be degraded. On the other hand, the propagation attenuation of higher frequency ultrasound is strong, and it is difficult to obtain recognizable receiving signals.

The resonance frequency of a circular MEMS ultrasonic transducer with different diameters is simulated by the equivalent circuit model and finite element analysis (FEA). Figure 1e shows that when the thickness *t* and the cavity depth *h* are constant, the frequency decreases as the diameter increases and is inversely proportional to the square of the diameter. The frequency varies from 2.18 to 0.81 MHz when the diameter changes from 80 to 130 μm. When the diameter is 120 μm, the frequency from the equivalent circuit model is 964.4 kHz, and the resonance frequency from FEA is 993 kHz. Therefore, the cavity diameter is selected to be 120 μm. The numerical values of the FEA (blue) and the equivalent circuit model (red) are slightly different. This is attributed to the addition of the upper electrode and passivation layer in the finite element simulation, and the parameters in the two models are also slightly different.

The relationship between the normalized displacement sensitivity (sensitivity ratio of all displacement data points to the maximum displacement) and the side length ratio of the upper electrode and the cavity is shown in Fig. 1f. When the diameter ratio of the upper electrode to the cavity is 0.707–0.72, the sensitivity of the ultrasound emission reaches the maximum. In this work, the ratio of the top electrode to the cavity selected in this paper is 0.71. The thicknesses of the bottom electrode, piezoelectric layer, top electrode and passivation layer are 0.5 μm, 0.5 μm, 0.15 μm and 0.1 μm, respectively.

### On-demand design of transducer array

When a transducer is used as an ultrasound receiver, it needs to be connected to an electrical circuit, including amplifiers and filters. Since the circuit has relatively large parasitic capacitances, which can result in the receiving voltage *V*_*p*_ being divided by the parasitic capacitances, the receiving voltage signal *V* becomes too small and is submerged in noise.

The capacitance of a single MEMS ultrasonic transducer is approximately 0.9 pF. In the whole test system, the external parasitic capacitance includes the PCB board capacitance *C*_*p*_ (3.2 pF), circuit connection line capacitance *C*_*l*_ (8 pF) and test circuit capacitance *C*_*e*_ (52.4 pF). The capacitance of each section was tested by an impedance analyzer (E4990A, Keysight Technologies, America). It can be clearly seen from Eq. (8) how much the signal voltage is reduced by parasitic capacitances.

8 $$V = \frac{{C_0}}{{C_0 + C_p + C_l + C_e}} \ast V_p$$

To reduce the influence of parasitic capacitance on the receiving performance, the transducers are connected in parallel to form a square array. As the number of arrays increases, the device capacitance increases, and the influence of parasitic capacitance weakens. The capacitance of the entire array is preferably 3 times greater than the parasitic capacitance to achieve a good receiving performance.

**Fig. 2:**
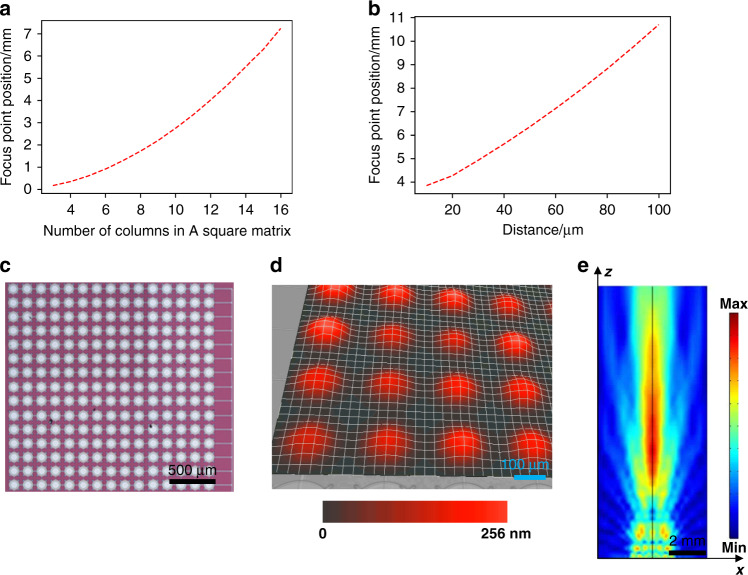
Design of the MEMS ultrasonic transducer array. **a** Relationship between the number of arrays and the focus point position. **b** Relationship between the element spacing and focus point position when both the number of rows and number of columns are 15. **c** A 15 by 15 transducer array with an element pitch of 170 μm. **d** The vibration mode of the MEMS ultrasonic transducer array. **e** Normalized distribution of acoustic emission intensity by the MEMS ultrasonic transducer array

As the number of transducers increases, the generated sound pressure also increases, even though the transducer area of the entire array becomes larger. The near and far fields of the acoustic field are related to the area, element arrangement and acoustic wavelength. Therefore, the near and far fields can be adjusted by changing the area of the array, which is mainly associated with the number of transducers and the spacing between individual devices. Figure 2a shows the relationship between the number of transducers and the focus point position when the device center distance is 170 μm. The horizontal axis is the number of rows or the number of columns. When the number of arrays increases, the area of the array increases, and the focus point position increases accordingly. When the number of elements increases from 3 by 3 to 16 by 16, the focus point position increases from 0.17 mm to 6.3 mm. Figure 2b shows the relationship between the element spacing and the focus point position when the element number of the array is 15 by 15. As the spacing increases, the focus point position also changes significantly. When the spacing increases from 10 μm to 100 μm, the focus point position increases from 3.85 mm to 10.7 mm accordingly.

As mentioned above, the general distance from the eye to the glasses is approximately 9 mm to 12 mm. Considering that the distance will be further reduced when the eyes are closed, the near field distance is best set at less than 7 mm. Therefore, considering these factors, such as parasitic capacitance, near-field distance and process level, we selected a 15 by 15 transducer array with an element pitch of 170 μm. The MEMS ultrasonic transducer array is shown in Fig. 2c. The theoretical capacitance of the array is 202.5 pF, which is greater than the parasitic capacitance of the rest of the system. The effective area of the MEMS ultrasonic transducer array is 2.5 mm by 2.5 mm. The weight of the MEMS ultrasonic transducer is 23.3 mg. The vibration mode of the MEMS ultrasonic transducer array, which is measured by a laser Doppler vibrometer (MSA600, Polytec, Germany), is shown in Fig. 2d. The excitation signal is a continuous wave with a voltage of 5 V_pp_ and a frequency of 960 kHz. The displacement sensitivity is 51 nm/V, as the displacement is 256 nm when the excitation voltage is 5 V_pp_. The emitted sound beam of the array which is simulated by FEA in Fig. 2e has good focusing, and the distance of the focal point is 6.35 mm away from the transducer surface, which meets the needs of the subsequent blinking test.

### Fabrication

**Fig. 3:**
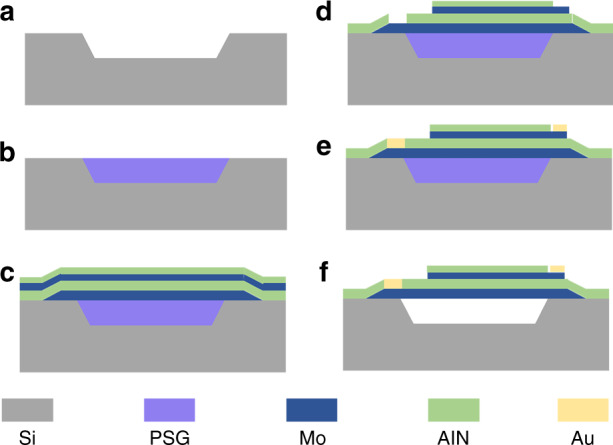
MEMS fabrication flow of the transducer element. **a** Etching cavity. **b** Deposition and polishing PSG. **C** Deposition and patterning of bottom electrode, and deposition of piezoelectric layer, top electrode and passivation layer. **d** Patterning of passivation layer, top electrode and piezoelectric layer. **e** Deposition and patterning of gold. **f** Etching PSG to form the cavity.

The transducer was fabricated by the MEMS process. First, a 3.5 μm depth cavity was etched on a 400 μm thick silicon wafer, as shown in Fig. 3a. Then, the cavity was filled with phosphosilicate glass (PSG), and after polishing the PSG, the depth of the cavity and the thickness of the PSG were 3 μm (Fig. 3b). The Mo bottom electrode was first deposited by sputtering onto the silicon wafer and the PSG, and then the Mo layer was patterned. After that, the AlN piezoelectric layer, Mo top electrode and AlN passivation layer were deposited by successive sputtering (Fig. 3c). The top electrode and the passivation layer were etched, and then the piezoelectric layer was patterned (Fig. 3d). A gold layer was deposited on part of the top electrode and bottom electrode as connection pads (Fig. 3e). Finally, a hydrofluoric acid solution was used to release the device and form an air cavity under the suspended structure (Fig. 3f).

### Electrical and acoustic characterization

**Fig. 4:**
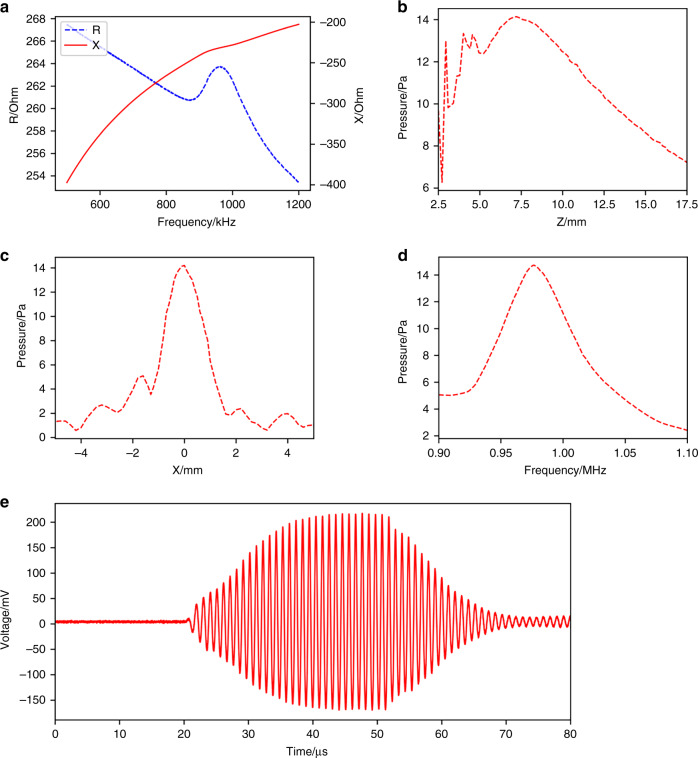
Electrical and acoustic characterization of the MEMS ultrasonic transducer array. **a** Measured electrical impedance of the MEMS ultrasonic transducer array with a resonance frequency of 960 kHz. **b** Measured axial sound pressure of the MEMS ultrasonic transducer array. **c** Measured lateral pressure directivity of the MEMS ultrasonic transducer. **d** Measured acoustic pressure response of the MEMS ultrasonic transducer array at different frequencies. **e** Time domain response at the focal point position (7.2 mm) of the MEMS ultrasonic transducer array

The electrical characteristics of the MEMS ultrasonic transducer array were measured by an impedance analyzer, and the test result is shown in Fig. 4a. The resonant frequency of the MEMS ultrasonic transducer array is 960 kHz, which is basically consistent with the above design. The slight difference may be caused by the fabrication residual stress in the structure films.

An optical microphone (Eta250, XARION Laser Acoustics GmbH, Austria) was used to evaluate the emitted acoustic pressure of the MEMS ultrasonic transducer array. The sensitivity of the optical microphone is 9.7 mV/Pa when the detection band ranges from 10 Hz to 1 MHz. The axial acoustic pressure distribution of the array is shown in Fig. 4b. The normalized pressure is the ratio of all sound pressure data points to the maximum sound pressure. The focus point position is 7.2 mm. In the near field range, the sound pressure changes irregularly. Therefore, the near field range is generally not used in application. In the far field range, as the distance increases, the sound pressure decreases. The maximum displacement from the eye to the device is approximately 12 mm, where the sound pressure at this distance is approximately 0.77 times that of the focus point position, which does not cause much decrease in the sound pressure. When the distance is as far as 1.8 cm, the sound pressure is half of the focus point position, which can fully meet the monitoring requirements of blinking.

Figure 4c shows the lateral acoustic pressure directivity of the MEMS ultrasonic transducer array. The −6 dB sound beam bandwidth is 1.7 mm, which has good acoustic pressure directivity. The slight asymmetry between the left and right parts is because the frequencies of the entire array elements are not completely consistent, which results in different start-ups and measurement errors. Even if the resonance frequency of the device is slightly uneven, it has no obvious influence on the acoustic pressure and the acoustic signal. Figure 4d shows the acoustic pressure of the MEMS ultrasonic transducer array at different drive frequencies. The −6 dB bandwidth of the frequency ranges from 938 kHz to 1020 kHz.

Figure 4e shows the time domain acoustic pressure signal at the focus point position of the MEMS ultrasonic transducer array when applying 30 cycles with an input of 20 V_pp,_ which ensures that the device achieves a complete vibration state. Note that the measured acoustic pressure at the focal point position is 14.57 Pa, corresponding to the spatial-peak temporal average *I*_*SPTA*_ of 0.48 μW/mm^2^ even with continuous wave excitation. When pulse waves with 5 cycles and a pulse repetition interval (PRI) of 10 ms are used, the *I*_*SPTA*_ is smaller than 0.24 nW/mm^2^ as the number of cycles decreases and the sound pressure decreases, meeting the requirements of the Food and Drug Administration (FDA) guidance for Marketing Clearance of Diagnostic Ultrasound Systems and Transducers (0.17 mW/mm^2^)^24^. Therefore, there should be no harm to the body^25,26^, guaranteeing that the transducer does not damage the eyes.

## Working principle and system setup

**Fig. 5:**
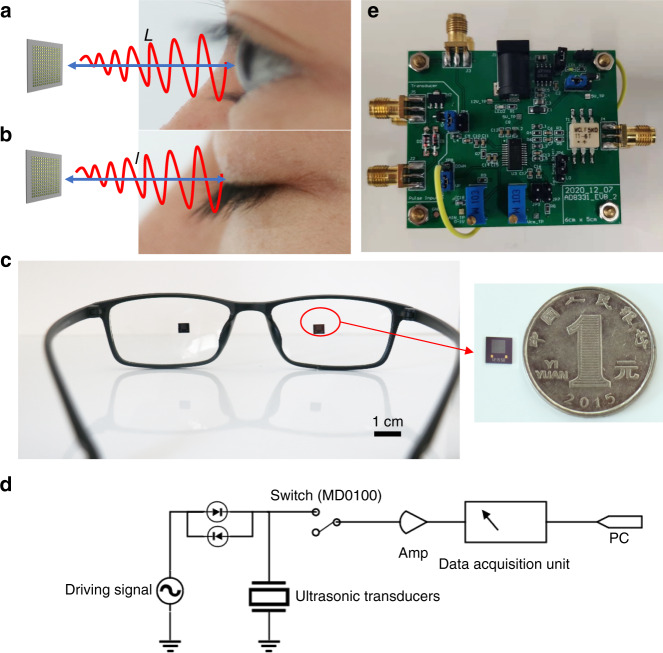
Working principle and system setup. **a** Schematic of ultrasonic wave propagation when eyes are open. **b** Schematic of ultrasonic wave propagation when eyes are closed. **c** Two MEMS ultrasonic transducer arrays integrated into glasses. **d** Schematic diagram of the test circuit. **e** Photo of the test circuit board

The MEMS ultrasonic transducer is electrically excited in air, vibrating due to the inverse piezoelectric effect and emitting ultrasonic waves. When the ultrasound encounters the eyes or eyelids, most of the ultrasound is reflected and returns to the MEMS ultrasonic transducer along the original path, as shown in Eq. (9), because the acoustic impedance of the eye is much greater than that of the air. Due to the piezoelectric effect, the MEMS ultrasonic transducer generates a receiving signal when subjected to ultrasonic vibration. As shown in Fig. 5a, when the eyes are open, the ultrasonic wave is reflected by the eyeball surface, and the distance of wave propagation before reflection is *L*. When the eyes are closed, as shown in Fig. 5b, the ultrasonic wave is echoed after encountering the eyelid, and the distance of wave propagation before reflection is *l*, which differs from *L* because of the thickness of the eyelids. As a result, the propagation time is also different. By comparing the TOF of the pulse echo, we can differentiate the states of open and closed eyes. The time difference can be given by Eq. (10).

9 $$T = \frac{{Z1 - Z2}}{{Z1 + Z2}}$$

10 $$t = \frac{{2(L - l)}}{c}$$

where *T* is the reflectivity, *Z1* is the acoustic impedance of the obstacle, and *Z2* is the acoustic impedance of the air.

The experimental device is shown in Fig. 5c, and the PCB board with a MEMS ultrasonic transducer is fixed to the glasses. The MEMS ultrasonic transducer array is driven by five sinusoidal pulses with a peak-to-peak value of 20 V, and the pulse repetition interval (PRI) is 10 ms. With the above driving condition, the power consumption of the transducer is 71 μW and is far less than the energy consumed by a camera, providing the possibility of extended use without battery charging. The schematic diagram and physical diagram of the test circuit are shown in Fig. 5d, e, respectively. The circuit can be divided into two parts: the transmitting driving part and the receiving part. The transmitting driving part uses a programmable signal generator, which generates the demanded pulse wave signal. The T/R switch (MD0100) is used to isolate the driving signal pulse from the echo signal. In the receiving part, the pulse echo signal is amplified by a differential amplifier (AD8331) and is collected by a USB data acquisition module (PICO Technology). The collected signal is processed and converted into a digital signal. In addition, an 8-order Butterworth bandpass digital filter is designed to filter out the clutter and process the digital signal. Its lower cutoff frequency is 0.251 MHz, and the upper limit is 510 MHz.

## Results and discussion

### Eye-blinking test in open and closed states

Using the above system, we tested three different blinking states: one single eye open or closed, two eyes with both open or closed, and two eyes with one open and the other closed.

#### One single eye open or closed

**Fig. 6:**
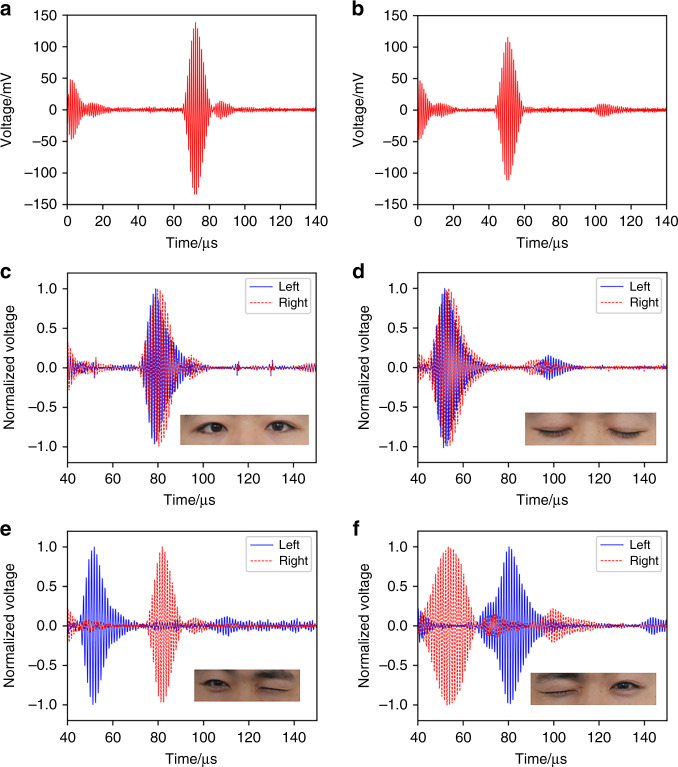
Static test of eye blinking. **a** The pulse echo diagram when one eye is open. **b** The pulse echo diagram when the eye is closed. **c** The pulse echo diagram when the two eyes are open. **d** The pulse echo diagram when two eyes are closed. **e** The pulse echo diagram when the left eye closed and the right eye opened. **f** The pulse echo diagram when the right eye closed and the left eye opened

Figure 6a, b shows the ultrasound pulse-echo signals at the states of one single eye open and closed, respectively. The transducer receives an input electrical signal of 20 V_pp_, 5 cycles, and a pulse repetition interval (PRI) of 10 ms. A Hilbert transform is performed on the receiving signal to obtain the envelope, and the maximum value time of the echo signal curve is taken as the characteristic time. The frequency is 960 kHz, so the ultrasonic wavelength is 357 μm, and the longitudinal resolution is 893 μm. In the eyes open state (Fig. 6a), the maximum value time and start-up time of the pulse echo are 72 µs and 66 µs, respectively. The V_pp_ of the receiving voltage is approximately 270 mV, and the signal-to-noise ratio is 30.6 dB as the system noise is 8 mV. In comparison, in the eyes closed state (Fig. 6b), the maximum value time and start-up time of the pulse echo are 51 µs and 44 µs, respectively. The receiving voltage is approximately 230 mV with a signal-to-noise ratio of 29.2 dB. The measured signal-to-noise ratio meets the requirements of feature time extraction in subsequent monitoring demonstrations.

Based on the sound velocity and the start-up time, the travel distances in the eyes open and closed states are calculated to be 7.55 mm and 11.15 mm, respectively. They are at the far field of our devices since the focus point position is 7.2 mm. The start-up time of the pulse echo is only used to calculate the distance, and the TOF is the time corresponding to the maximum of the envelope unless otherwise specified.

Interestingly, it seems that the receiving signal should increase due to a reduction in distance when the eye is closed. However, our measurement shows that the signal is reduced when the eyes are closed. This may be due to a coarser surface of the eyelid than that of the eyeball.

#### Two eyes with both open or closed

Figure 6c, d shows the ultrasound pulse-echo signals at the states of two eyes open and closed, respectively. Meanwhile, pulse-echo signals in Fig. 6c, d are normalized for clarity. When two eyes are open, the pulse echo TOFs of the left eye and the right eye are 79 µs and 80 µs, respectively. When two eyes are closed, the pulse echo TOFs of the left eye and the right eye are 51 µs and 53 µs, respectively. Therefore, the TOF differences between open and closed states for the left and right eyes are 28 µs and 27 µs, respectively. By extracting the TOF difference, the state of each eye could be determined. When eyes are closed, the difference in two eyelid muscle shapes may contribute to the distance discrepancy between the transducers and the eyelids. Therefore, we believe it contributes to the slight discrepancy in the TOF differences between the two eyes. However, the discrepancies in the eyelid shapes and corresponding TOF differences are small, and they have little impact on the blinking monitoring test.

#### Two eyes with one open and the other closed

Figure 6e shows the ultrasound pulse-echo signals when the left eye is closed and the right eye is open, and Fig. 6f shows the ultrasound pulse-echo signals when the left eye is open and the right eye is closed. For the first case, the pulse echo TOF of the left eye is 53 µs, and the pulse echo TOF of the right eye is 82 µs. Therefore, the TOF of the right eye is 29 µs behind that of the left eye, which is caused by the closed left eyelid. For the latter case, the pulse echo TOF of the left eye is 80 µs, and the pulse echo TOF of the right eye is 56 µs. Therefore, the TOF of the left eye lags behind that of the right eye by 24 µs, which is caused by the closed right eyelid. The time difference between the two states is different (29 µs vs. 24 µs), probably because the eyelid muscle strength is different when the eye is closed, resulting in a different eyelid shape and thus TOF discrepancy. Again, this should have little impact on the blinking monitoring test.

### Test results from different volunteers

**Fig. 7:**
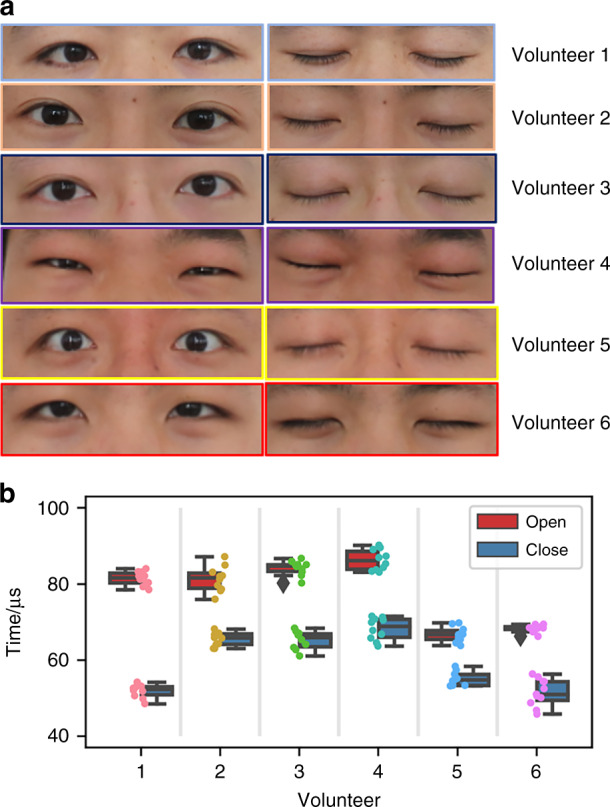
Eye-blinking test on six volunteers. **a** Photos of six subjects’ eyes when open and closed. **b** TOF of six subjects with eyes open and closed

To verify the universality of our system, we tested blinking activities on six people. Figure 7a shows photos of the eyes of our six subjects. Everyone’s eyes are different in size, degree of myopia, pupil distance and eyelid thickness. For example, the eyes of volunteer 4 are smaller than the others, and the eyes of volunteer 5 are nearsighted. For fair comparison, the distance between glasses and forehead is carefully calibrated for each subject. Figure 7b shows the difference in pulse echo TOF when the six people’s eyes were open and closed. When open, volunteers 1, 2, 3 and 4 have similar TOFs, approximately 80 µs; TOFs of volunteers 5 and 6 are approximately 65 µs. When closed, volunteers 2, 3 and 4 have similar TOFs, approximately 65 µs; TOFs of volunteers 1, 5 and 6 are approximately 50 µs. Interestingly, the TOFs of volunteers 5 and 6 with open eyes were roughly the same as the TOFs of volunteers 2, 3 and 4 with closed eyes. This is partially because the shapes of the human heads and eyes are different; in particular, there are symptoms of bulging eyes because of myopia. As the degree of myopia increases, the extent of bulging is more distinct, and the propagation distance of the pulse echo decreases, resulting in a drop in TOF. Similarly, TOF will also be affected by the different positions of glasses in different people’s experiments due to the different head shapes.

In this test experiment, the smallest TOF difference is 8.5 µs, and the largest TOF difference is 32.5 µs with the other four data in between. The discrepancy in TOF differences among different people is caused by the difference in their eyelid thicknesses, and the difference between double and single eyelids will also affect the value. Volunteer 1 had a double eyelid, volunteers 5 and 6 had a single eyelid, and the TOF difference caused by a double eyelid was slightly larger than that caused by a single eyelid. The eyelid of volunteer 4 was slightly thicker, and although it was also a single eyelid, the TOF difference was greater than that of volunteer 5. Therefore, it is feasible to individually determine the blinking activity state by the TOF change of ultrasonic pulse echo for each person.

From the pulse echo TOF results of the six subjects, it can be seen that the TOF difference of open and closed eyes for each person is different. Therefore, in the subsequent blinking monitoring application experiment, it is necessary to calibrate the TOF of each person’s open and closed eyes to calculate the threshold value for eye state determination. The process is similar to prerecording a fingerprint into a smartphone before any biometric application.

Due to the size and shape of each person’s eyes, when testing volunteer 4 and volunteer 6, the device was slightly adjusted on the glasses to ensure that the device could better align with the pupil. The other four had the same device location. For each volunteer in the test, the TOF changed slightly from time to time when their eyes were open from a closed state. This is caused by a slight movement of the eyes and a change in the position of the glasses. The TOF of closed eyes also varies due to possible differences in muscle morphology and changing position of the glasses each time the eyes are closed. In this work, the connection between the MEMS ultrasonic transducer arrays and the circuit board will exert an unwanted drag force on the glasses, which changes the original position of the glasses. This negative effect can be avoided when the discrete electrical circuit board is replaced by integrated circuit (IC) chips and integrates the entire system into the frames of the glasses. In our daily lives, eyeglasses are generally firmly held when worn, so they have very limited displacement relative to our heads.

### Real-time monitoring

The previous experiments were all carried out in a static process. However, in practical applications, it is generally a dynamic process. Therefore, we conducted a real-time monitoring experiment of eye blinking states. There were four states in the experiment: the left and right eyes were open at the same time; the left and right eyes were closed at the same time; the left eye was open and the right eye was closed; and the left eye was closed and the right eye was open.

**Fig. 8:**
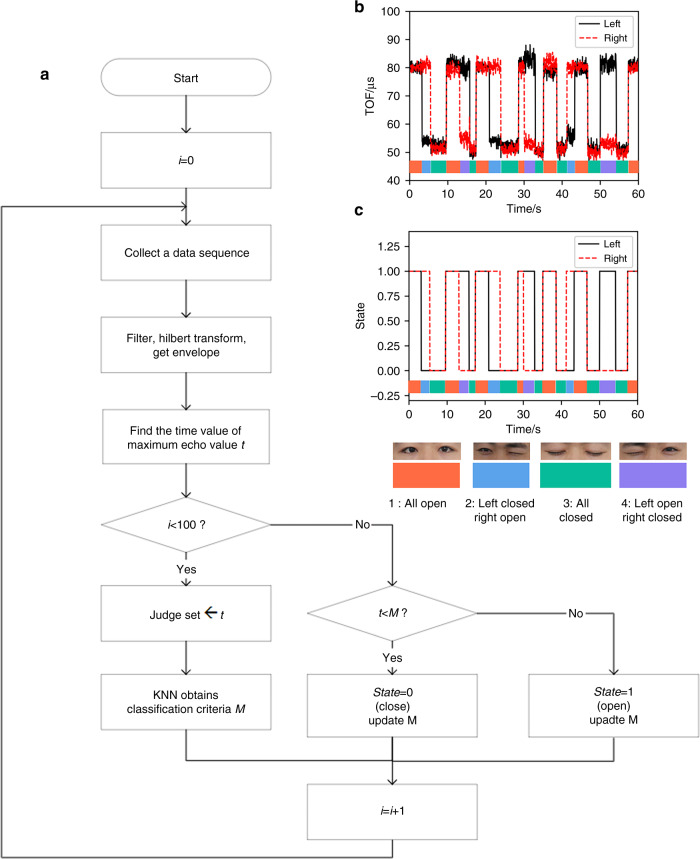
Real-time monitoring of eye blinking in one minute. **a** Algorithm diagram for data extraction. **b** Real-time measured signal of eye-blinking state monitoring for one minute. **c** State classifications for real-time eye-blinking monitoring by the K-means clustering method

The algorithm architecture of the monitoring procedure is shown in Fig. 8a. First, it collects data blocks for a period of time and filters and transforms them by the Hilbert method. Next, the envelope of the echo signal is obtained. Then, it finds the event corresponding to its maximum value as the TOF and uses the magnitude of this time value to determine the state. To better adapt to different people, a dynamic unsupervised learning method is used for state classification. The first 100 sets of data at the beginning of the experiment are divided into two clusters, which are regarded as labeled data. The midpoint of the two cluster centers is used as the criterion to classify the subsequent data. After that, the classified data are added to the cluster, the cluster center is recalculated, and the criteria are updated. Compared with supervised learning, this method does not need to label large amounts of data. At the same time, the calculation amount is greatly reduced, and the response time is less than 1 ms, which is more conducive to the real-time application of dynamic eye blinking monitoring.

Unlike the static test, the dynamic test may be affected by eyelashes. During the process of eye closure, there is a period of open state at the beginning, a period of closed state in the end, and a transition period in between. Since the blinking speed of humans is usually greater than 0.2 s each time, given that the pulse repetition interval in this work is 10 ms, there will be at least 20 sets of data in a transition process in the dynamic test of blinking. Eyelashes will reduce ultrasound travel distance, and therefore, their movement will dynamically change TOF values. Therefore, data influenced by eyelashes in this transition period are considered noise and removed from the real-time measurement signal.

Figure 8b shows the dynamic TOF values in one minute. The real-time blinking state can be easily seen in the figure. For example, the left eye is closed and the right eye is open from 3.6 to 5.8 s, the two eyes are closed from 5.8 to 9.8 s, the two eyes are open from 9.8 to 13.6 s, and the left eye is open and the right eye is closed from 13.6 to 15.9 s. It should be noted that the TOF of both closed eyes and TOF of one closed eye when the other is open are different. This is due to different muscle morphologies when both eyes are closed compared to when only one eye is closed. K-means clustering is performed on the first 100 groups of TOF data obtained from the experiment, and the threshold is 67 µs. The result of classifying the data are shown in Fig. 8c. The difference in muscle states does not affect our judgment and classification because the TOF change is significant when the eyes are closed from the open state.

## Discussion

The circuit used in this work is relatively large and heavy, and there are issues such as blocking the sight of volunteers and long-term testing for further optimization and development. The test circuit has a relatively large parasitic capacitance, which reduces the receiving signals of the transducer array. The parasitic capacitance should be further reduced. A transducer array can be integrated with a CMOS circuit, which can not only largely reduce the volume and weight of the test circuit but also reduce the parasitic capacitance of the circuit. This reduces the required number of elements in the transducer array and thus the volume of the whole system. With the integrated CMOS circuit and reduced device volume, more transducer arrays can be integrated into multiple locations of the glasses frame, thus avoiding blocking the sight of volunteers, and more fine-grained information about eye activity can be acquired by data from multiple sensors. Using wireless communication, the collected data can be updated to the user’s smartphones or servers for powerful data analysis.

Phased array control can be used to change the sound field directivity or focus point position. Without phase control, the main lobe of the sound field propagates axially along the transducer array. When the transducer array is fixed to the glasses frame perpendicularly, the main lobe of the sound field does not face the eye directly, so much of the sound wave could not reach the eyeball or eyelid position, and the receiving signal would be weak. Phased array control can be used to steer the directivity of the sound field so that the main lobe of the sound field faces directly to the ocular position. At the same time, phased array control can change the focus point position and improve the emission sensitivity, thus improving the detection performance.

In the current detection system, the driving voltage is 20 V_pp_. After adoption of phased array control and integration with the CMOS circuit, the transducer circuit noise decreases in addition to emission sensitivity increase and parasitic capacitance reduction. Therefore, the driving voltage and power consumption can be reduced to further meet the requirements of portable applications.

## Conclusion

This paper demonstrates the feasibility of ultrasound-based eye-blinking detection and monitoring using a portable device. Small, lightweight and low-power transducers are the key to realizing the portable pulse echo TOF ranging principle. Air-coupled 960 kHz MEMS ultrasonic transducer arrays were designed and fabricated, which measured 2.5 mm by 2.5 mm in size, 23.3 mg in weight and 71 μW of power consumption. In a pulse echo characterization experiment, the measured signal-to-noise ratio reached approximately 30 dB. Three static states were differentiated, including one eye open and the other closed, two eyes open and two eyes closed at the same time. For 6 different people, the TOF difference due to different blinking states was different, with a range from 8.5 µs to 32.5 µs. Finally, a dynamic eye blinking monitoring experiment was conducted in one minute, and the blinking state could be tracked in real time with a dedicated algorithm and a response time less than 1 ms. The solution we proposed has advantages in terms of portability, information security, biological safety, and reliability for eye blinking-related applications.

## Materials and methods

### Circuit design

An MD0100, a high-voltage, two-terminal, bidirectional and current-limiting protection chip, is chosen as the duplexer. The MD0100 chip is available in the SOT-89 package and has a typical switching resistance of 15 ohms, which allows weak signals to pass. Meanwhile, it avoids complex circuits because of its automatic switching control. These features are ideal for miniaturized ultrasonic applications.

An AD8331 is chosen as the amplifier chip. The AD8331 is a single-channel and ultralow noise amplifier that is optimized for ultrasonic systems. This chip includes an ultralow noise preamp (LNA), a variable gain amplifier (VGA) with a 48 dB gain range, and a selectable gain postamp with adjustable output limiting. It has excellent bandwidth uniformity across the required frequency range.

### Transducer fixed on glasses edge without blocking sight

**Fig. 9:**
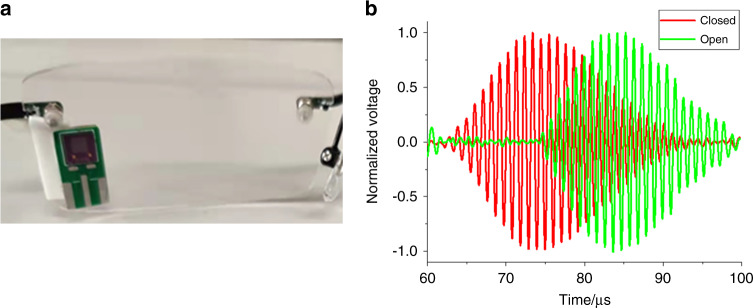
Transducer fixed on the glasses edge. **a** Optical photo for demonstration. **b** TOF change when the eye is open or closed

The transducer is fixed on the edge to avoid blocking the sight as much as possible, as shown in Fig. 9a. The experimental results are shown in Fig. 9b. The TOF change could be clearly seen when the eye is open or closed, which can distinguish between the states of open and closed. In the current prototype stage, the sizes of the transducer array and circuit are both relatively large. However, after subsequent integration of a transducer array and a CMOS circuit in the future, the integrated chip would be miniaturized, enabling its seamless integration into the glasses frame without completely blocking the sight.
